# Molecular and Histological Effects of Glyphosate on Testicular Tissue of the Lizard *Podarcis siculus*

**DOI:** 10.3390/ijms23094850

**Published:** 2022-04-27

**Authors:** Mariailaria Verderame, Teresa Chianese, Luigi Rosati, Rosaria Scudiero

**Affiliations:** 1 Department of Human, Philosophic and Education Sciences (DISUFF), University of Salerno, 84084 Fisciano, Italy; mverderame@unisa.it; 2 Department of Biology, University Federico II, Via Cintia 21, 80126 Napoli, Italy; teresianachianese@libero.it (T.C.); luigi.rosati@unina.it (L.R.); 3 Center for Studies on Bioinspired Agro-Environmental Technology (BAT), 80055 Portici, Italy

**Keywords:** glyphosate toxicity, spermatogenesis, estrogen receptor expression, connexin 43, steroidogenesis

## Abstract

The expansion of agriculture produces a steady increase in habitat fragmentation and degradation due to the increased use of pesticides and herbicides. Habitat loss and alteration associated with crop production play an important role in reptile decline, among which lizards are particularly endangered. In this study, we evaluated testicular structure, steroidogenesis, and estrogen receptor expression/localization after three weeks of oral exposure to glyphosate at 0.05 and 0.5 μg/kg body weight every other day in the field lizard *Podarcis siculus*. Our results show that glyphosate affected testicular morphology, reduced spermatogenesis, altered gap junctions and changed the localization of estrogen receptors in germ cells, increasing their expression; the effects were mostly dose-dependent. The result also demonstrates that glyphosate, at least at these concentrations, did not influence steroidogenesis. Overall, the data indicate that this herbicide can disturb the morphophysiology of the male lizard’s reproductive system, with obviously detrimental effects on their reproductive fitness. The effects of glyphosate must be considered biologically relevant and could endanger the reproductive capacity not only of lizards but also of other vertebrates, including humans; a more controlled and less intensive use of glyphosate in areas devoted to crop production would therefore be advisable.

## 1. Introduction

Currently, a major global challenge in agriculture is to increase production as much as possible to feed the ever-growing human population, using practices that are sustainable for the planet and, at the same time, able to obtain healthy products. Consequently, more and more attention has been paid to substances capable of increasing agricultural production, such as fertilizers, pesticides and herbicides. The latter, in particular, restrain the growth of weeds and favor the drying of crops such as wheat, making these crops easier and cheaper to produce [1,2]. In recent years, glyphosate (Gly) has been widely used; this chemical is a non-selective herbicide that is absorbed by the stem and leaves and then transported throughout the plant, blocking enolpyruvylshikimate-3-phosphate synthase (EPSPS), a key enzyme involved in the synthesis of aromatic amino acids essential for plant growth [3]. This inhibitory mechanism towards a missing enzyme in animal tissues and its rapid degradation made glyphosate the herbicide of choice for many years. Unfortunately, many studies have shown the ability of Gly to accumulate in plants treated or grown on treated soils [4,5], in water [6,7] and, from there, to pass into animal tissues through the food chain [6,8]. Gly has been found in human urine samples and at higher concentrations in populations living in countries where its use in agriculture is broader, such as the USA [9]. In addition to humans exposed professionally or through food, wildlife is particularly threatened by the action of Gly. Fish exposed to Gly-contaminated water show increased plasma glucose and cortisol levels, endocrine dysregulation, alterations in the antioxidant enzyme systems of hepatocytes and in transcriptome [10,11]; in amphibians, Gly causes teratogenic effects and developmental failures impacting both larval and adult stages [12,13,14]. DNA breaks, changes in immune parameters and plasma proteins, keratin disturbance and growth retardation have been observed in reptiles and birds exposed to Gly [15,16,17,18].

In recent years, greater attention has been paid to glyphosate-induced reproductive toxicity, especially in males, due to the growing interest in male reproductive toxicology because of the decline in human sperm quality worldwide [19,20,21].

It has been demonstrated that environmental pollution can produce a deterioration in human semen quality, altering spermatozoa morphology, vitality, count and motility but also causing DNA fragmentation and alterations in the protamines/histones ratio; all these adverse effects seem to be related to an imbalance in reactive oxygen species (ROS) [22]. An increase in transgenerational effects was also highlighted, which, over time, could influence the rapid decline in semen quality and endanger the survival of populations living in particularly polluted areas [22].

In rodents, Gly exposure causes reproductive disorders, including abnormalities in sperm viability and concentration [23,24,25], disruption of blood–testis barrier integrity [26,27] and testicular oxidative stress [23,28]. In vitro exposure of mouse MA-10 Leydig tumor cells to Gly or Gly-based herbicides (GBH) also affects the expression of the steroidogenic acute regulatory protein (StAR) [29], which mediates the initial step of steroid hormone synthesis [30], such as androgens, and reduces transcription and the activity of the enzyme aromatase [31], the key player in the aromatization of androgens to produce estrogens [32,33,34]. Adverse effects on the morphology of the seminiferous tubules are observed, such as a reduction in the epithelium and the enlargement of the lumen [35]. Luminal dilation and epithelial regression were also found in the testis of avian species exposed to Gly [36]. Similar testicular abnormalities have been detected following exposure to estrogen or xenoestrogenic substances [37,38,39,40]; this has led to the conclusion that Gly has estrogenic effects and therefore should be considered an endocrine-disrupting chemical (EDC) [41,42].

A group of wild animals particularly endangered by habitat loss and soil contamination is reptiles. Among them, lizards are an important link between invertebrates and higher predators; they represent the group of reptiles on which ecotoxicological studies have focused the most [43]. Prey to many small mammals and birds, they also play an important role as soil predators, contributing, in various areas, to the control of agricultural pests. Obviously, being part of the wildlife, they can be affected by contamination from anthropogenic activities [44,45,46,47,48].

Recently, we demonstrated that the oral administration of glyphosate, albeit at low doses, can induce hepatotoxic and estrogen-like action in the Italian field lizard *Podarcis siculus* [49]. Histological analysis showed the presence of fibrotic formations, as well as the induction of defense pathways from oxidative stress. Interestingly, the investigation, which was aimed at verifying the presence of the biomarkers of estrogenic contamination in the liver of male specimens, i.e., the presence in hepatocytes of vitellogenin and estrogen receptors transcripts, clearly demonstrated the xenoestrogenic action of glyphosate [49].

The aim of the present study was to investigate the effects of pure Gly on male reproductive tissues of the lizard *P. siculus*, the spermatogenic activity of which is well documented [50], identifying the histological and molecular effects related to its xenoestrogenic action. The possible alterations were evaluated by analyzing testis architecture and morphology, estimating the modifications of the spermatogenesis process, the disturbance of steroidogenesis and the modifications in the expression and localization of estrogen receptors. The data collected provide insight into the male reproductive toxicity of *P. siculus* induced by glyphosate.

## 2. Results

### 2.1. Morphological Analysis of Testis

**Figure 1:**
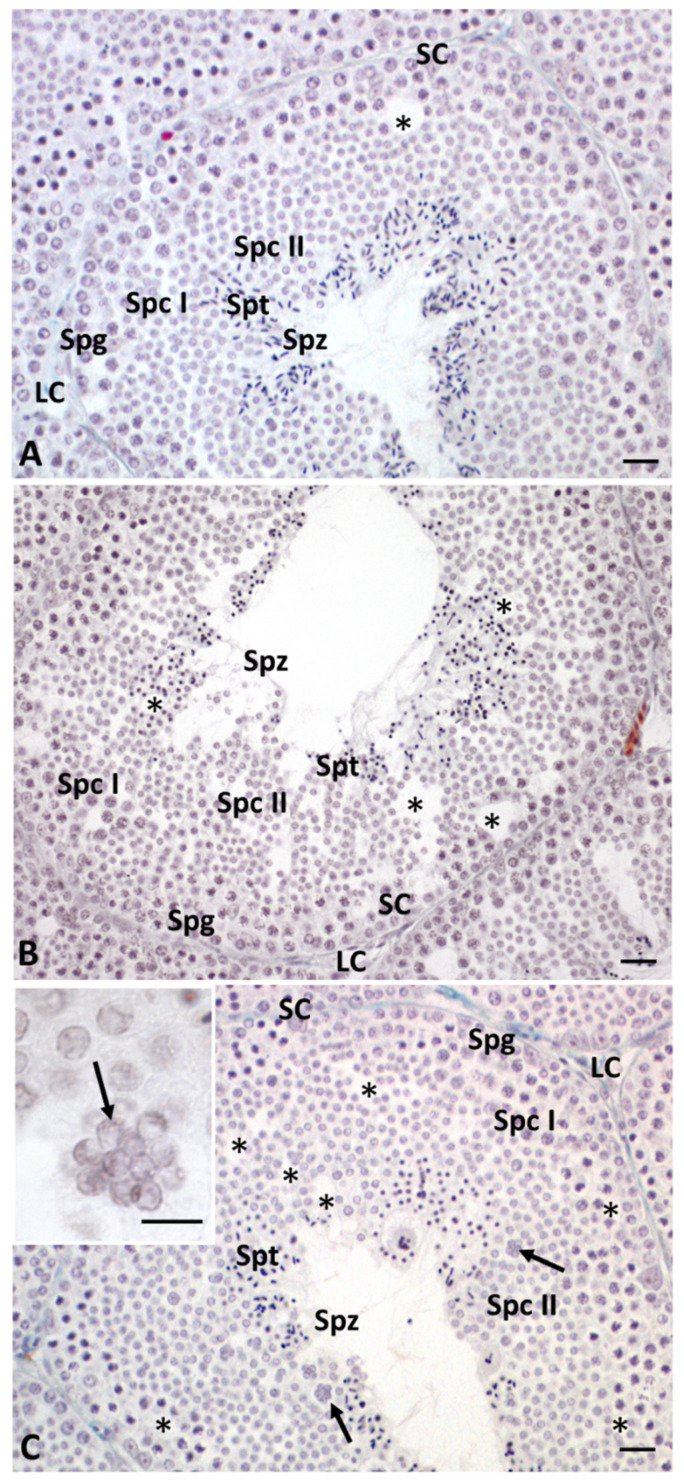
*P. siculus* testis stained with Mallory trichrome. (**A**) Testis control. (**B**) Testis from LD group animals. (**C**) Testis of HD animals. SC: Sertoli cells, LC: Leydig cells, Spg: spermatogonia, Spc I: spermatocytes I, Spc II: spermatocytes II, Spt: spermatids, Spz: spermatozoa, *: empty space between germ cells. Scale bars correspond to 20 µm.

The samples of the control group showed a testicular organization typical of the autumnal resumption period [50], whereby the seminiferous tubules were composed of Sertoli cells and germ cells in all phases of the spermatogenetic cycle, from spermatogonia (Spg) to spermatozoa (Spz); Leydig cells (LC) were present in the interstitial spaces (Figure 1A).

In testis of low-dose (LD)-treated animals, it was still possible to show the presence of germ cells in all stages of differentiation, but only a few spermatozoa were detectable in the lumen of the tubules (Figure 1B).

**Figure 2:**
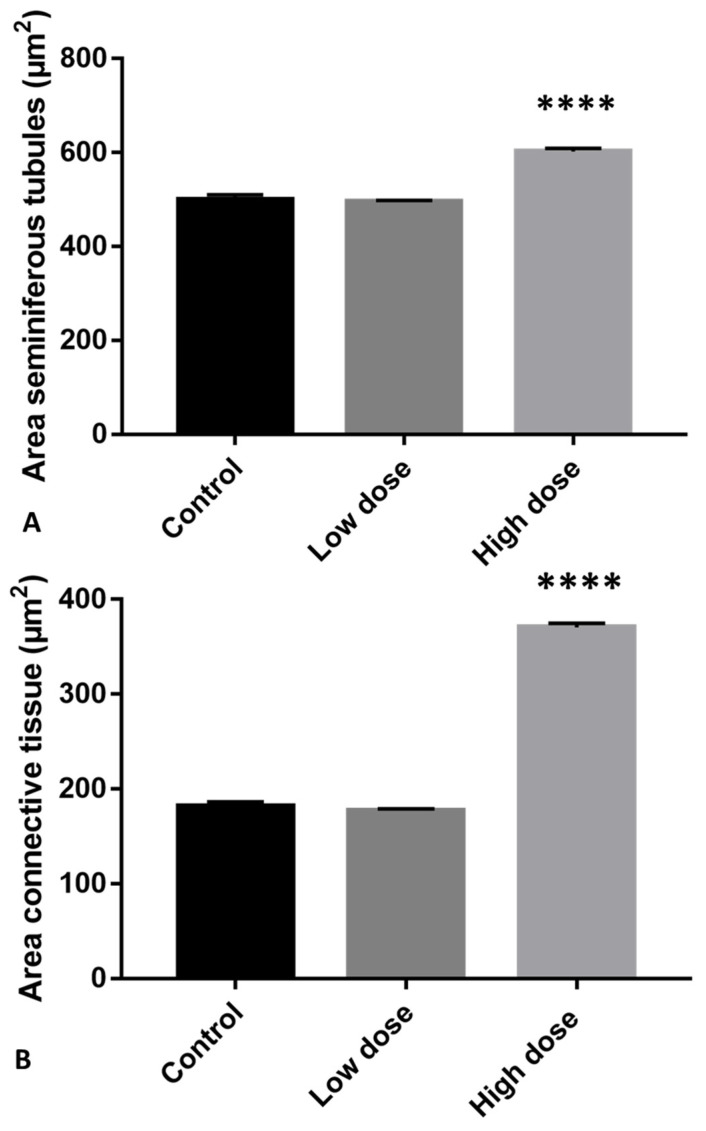
Morphometric analysis of *P. siculus* testis treated with different concentrations of glyphosate. (**A**) Area of seminiferous tubules. (**B**) Area of connective tissue. **** *p* < 0.0001.

Moreover, the seminiferous tubules from this group showed more germinal epithelial gaps than the control (Figure 1B). Treatment with the high dose (HD) of glyphosate resulted in an increase in both areas of connective tissue and seminiferous tubules with overlapping cells, as well as an increase in empty spaces between germ cells compared to the control and LD treatment. In the seminiferous tubules of HD-treated animals, we also found very few spermatozoa and the presence of rosette-shaped cell aggregates between the cells of the germinal epithelium (Figure 1C, Figure 2).

**Figure 3:**
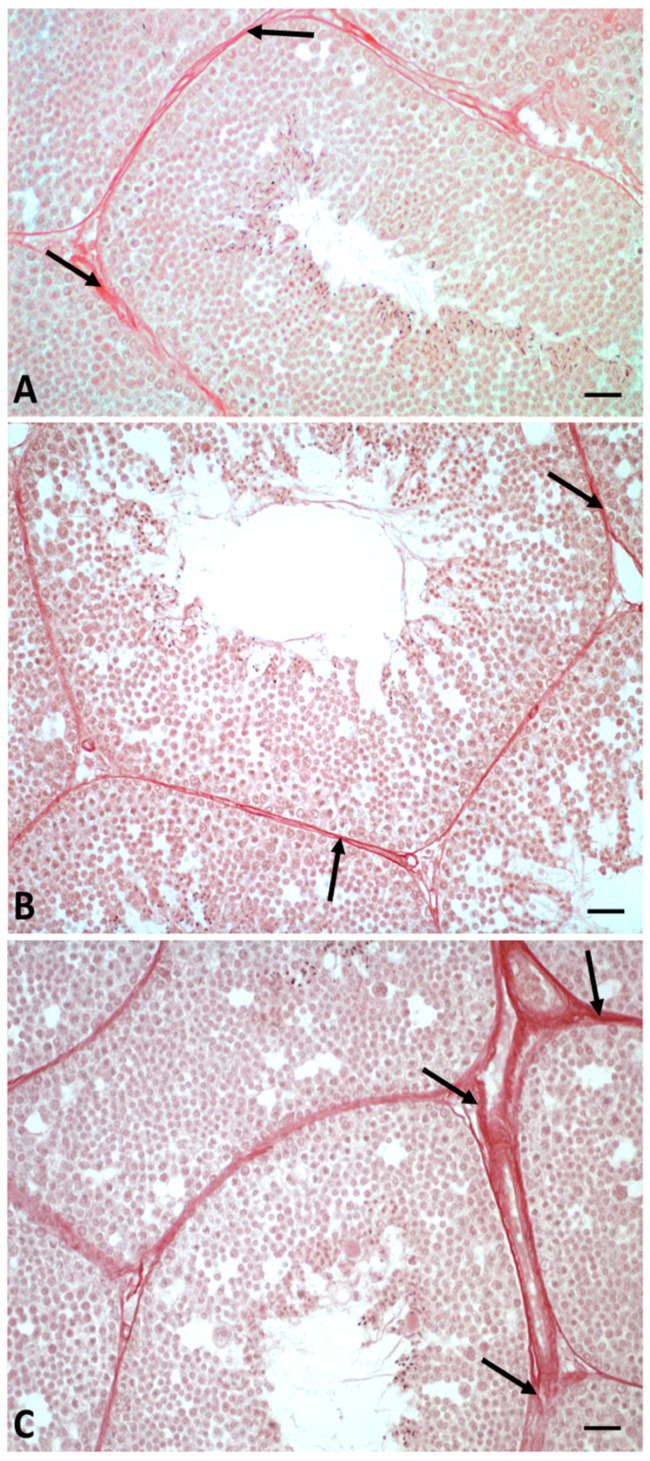
*P. siculus* testis stained with picrosirius red. (**A**) Testis control. (**B**) Testis from LD group animals. (**C**) Testis of LD animals. Arrow: collagen deposition (fibrosis). Scale bars correspond to 20 µm.

In addition, picrosirius red staining in this experimental group showed the deposition of fibrotic collagen in the interstitial spaces between seminiferous tubules, what was not detected in the control and LD-treated samples (Figure 3).

### 2.2. Immunohistochemistry for Cx43, Steroidogenic Enzymes, Erα and Erβ in Testis Sections

**Figure 4:**
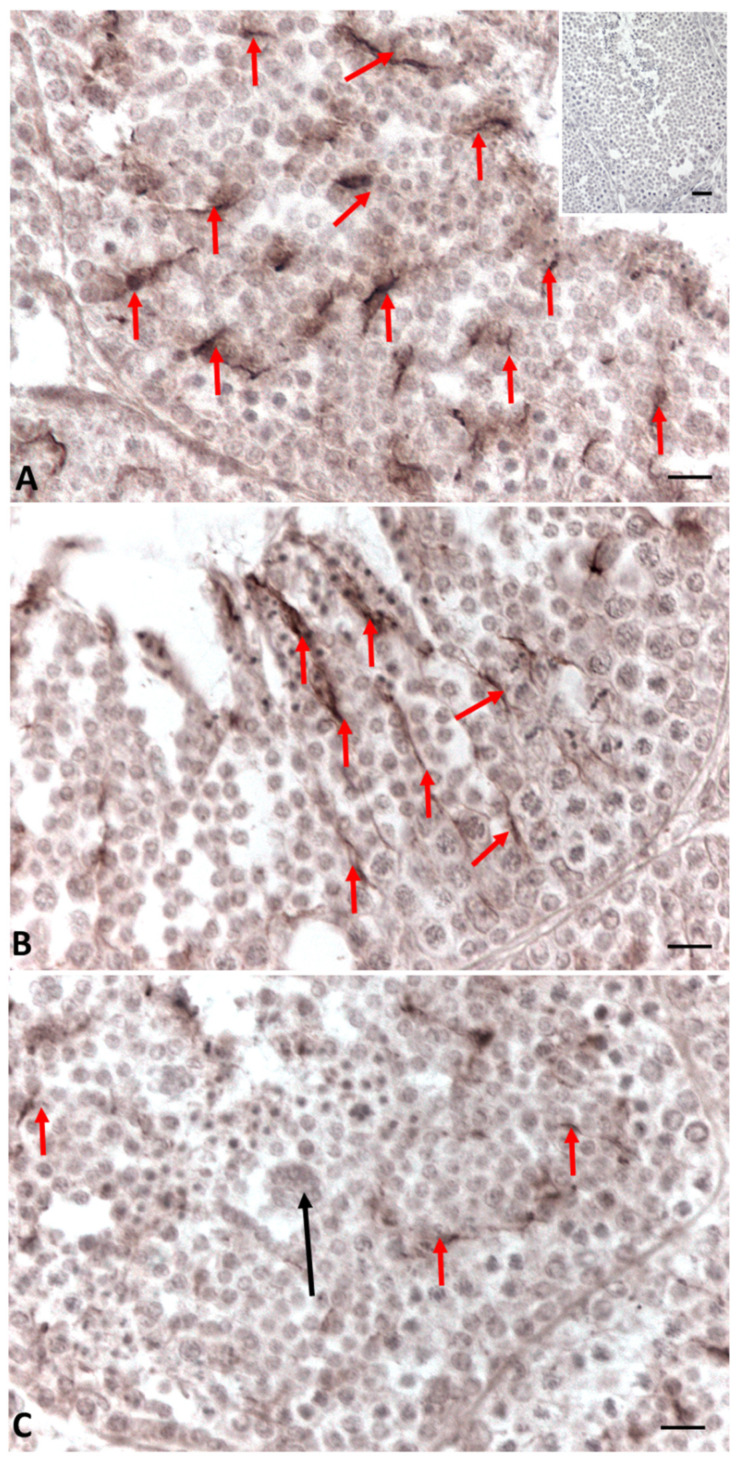
Immunohistochemical localization (brown areas) of Connexin 43 in testis of *P. siculus* treated with different concentration of glyphosate. (**A**) Control. The signal is evident along all the Sertoli cells (red arrows). (**B**) Low dose. Positivity is only evident along some Sertoli cells (red arrows). (**C**) High dose. (**A**): reduced immunopositivity is evident only in spots (red arrows). (**A**, insert): no signal is present in control sections. Black arrow: rosette-shaped cell aggregates. Scale bars correspond to 15 µm in figure (**A**–**C**) and 50 µm in figure (**A** insert).

Immunohistochemical analysis of Connexin 43 (Cx43), the predominant testicular gap-junction protein, showed that in control testes, the protein is present at all contact points between two Sertoli cells and between Sertoli cells and germ cells throughout the seminiferous tubule (Figure 4A).

The distribution of Connexin 43 was reduced in the group treated with the low dose of Gly, where Cx43 was present only in some areas of the seminiferous tubules (Figure 4B).

**Figure 5:**
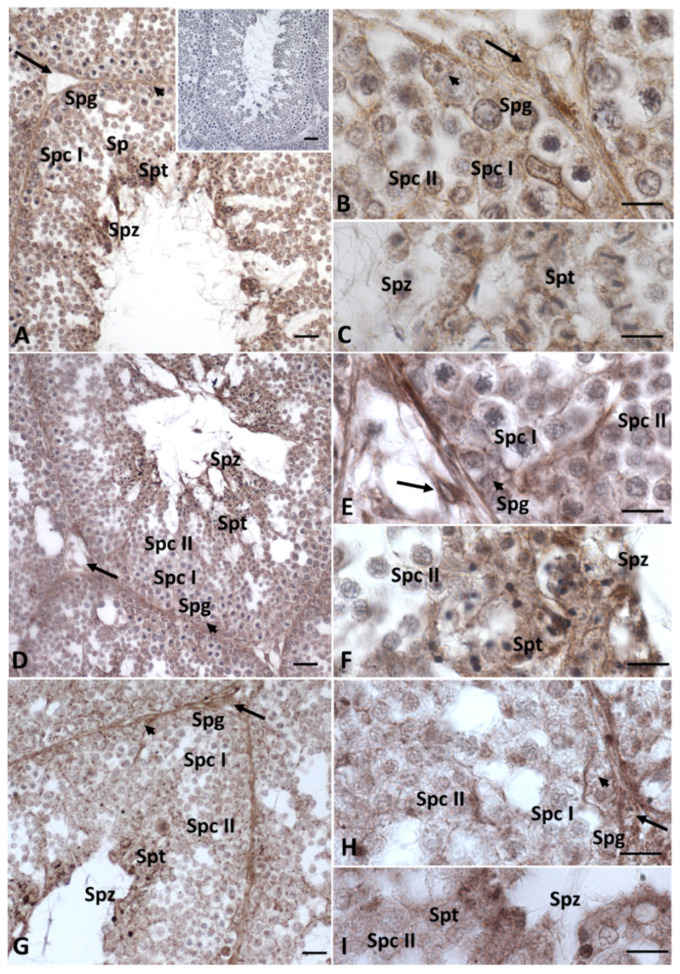
Immunohistochemical localization (brown areas) of P450 aromatase in testis of *P. siculus* treated with different concentrations of glyphosate. (**A**–**C**) Control. The signal is evident in Leydig (arrow) cells, spermatids (Spt), and spermatozoa (Spz); a faint signal occurs in Sertoli (arrowhead) cells and in spermatogonia (Spg). (**D**–**F**) Low dose. The enzyme is evident in Leydig (arrow) cells, spermatids (Spt) and spermatozoa (Spz); a faint signal occurs in Sertoli (arrowhead) cells and in spermatogonia (Spg). (**G**–**I**) High dose. Immunopositivity is evident in Leydig (arrow) cells, spermatids (Spt) and spermatozoa (Spz); a faint signal occurs in Sertoli (arrowhead) cells and in spermatogonia (Spg). (**A** insert): no signal is present in control sections. Scale bars correspond to 5 µm in figure (**B**,**C**,**E**,**F**,**H**,**I**) and 20 µm in figures (**A**,**D**,**G**) and 50 µm in figure (**A** insert).

**Figure 6:**
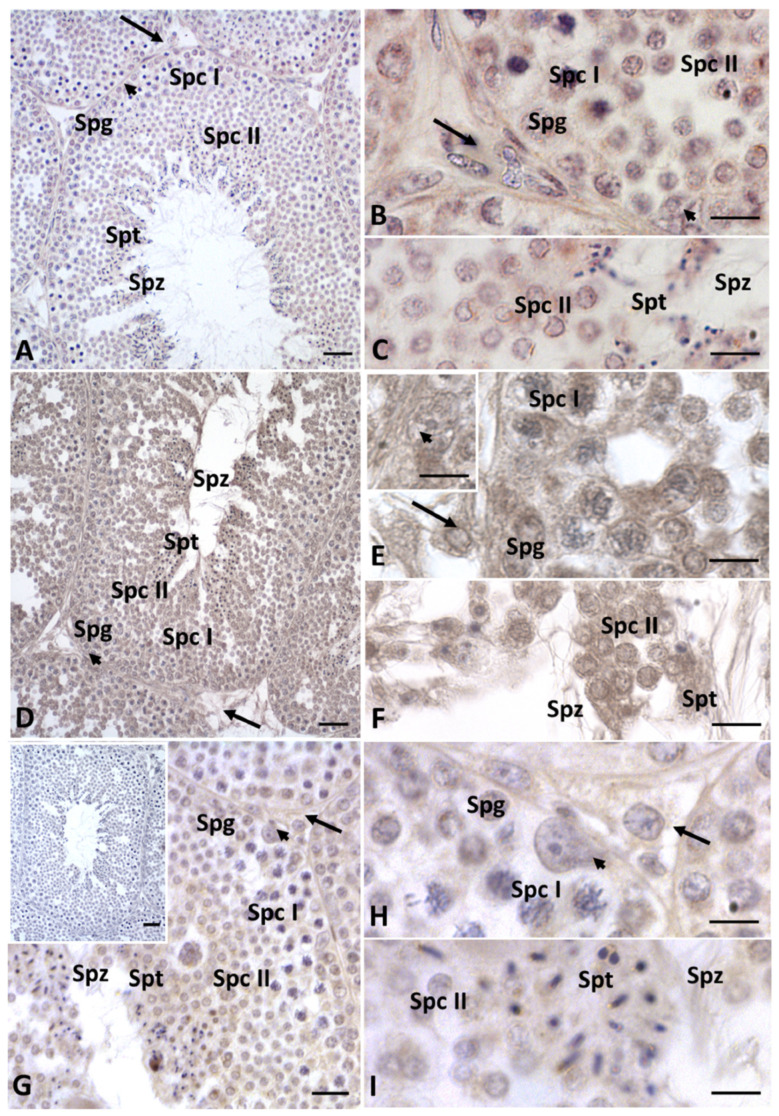
Immunohistochemical localization (brown areas) of ERα in testis of *P. siculus* treated with different concentrations of glyphosate. (**A**–**C**) Control. The ERα signal is evident in Sertoli cells (arrowhead), spermatocytes I (Spc I) and II (Spc II), spermatids (Spt) and spermatozoa (Spz). (**D**–**F**) Low dose. The signal is evident in in Leydig (arrow) cells and Sertoli cells (arrowhead), spermatocytes I (Spc I) and II (Spc II), spermatids (Spt) and spermatozoa (Spz); (**E** insert): detail of a Sertoli cell. (**G**–**I**) High dose. Positivity is evident in Leydig (arrow) and Sertoli cells (arrowhead), spermatocytes I (Spc I) and II (Spc II), spermatids (Spt) and spermatozoa (Spz). (**G** insert): no signal is present in control sections. Scale bars correspond to 5 µm in figure (**B**,**C**,**E**,**F**,**H**,**I**); 15 µm in figure (**G**); 20 µm in figures (**A**,**D**); and 50 µm in figure (**G** insert).

**Figure 7:**
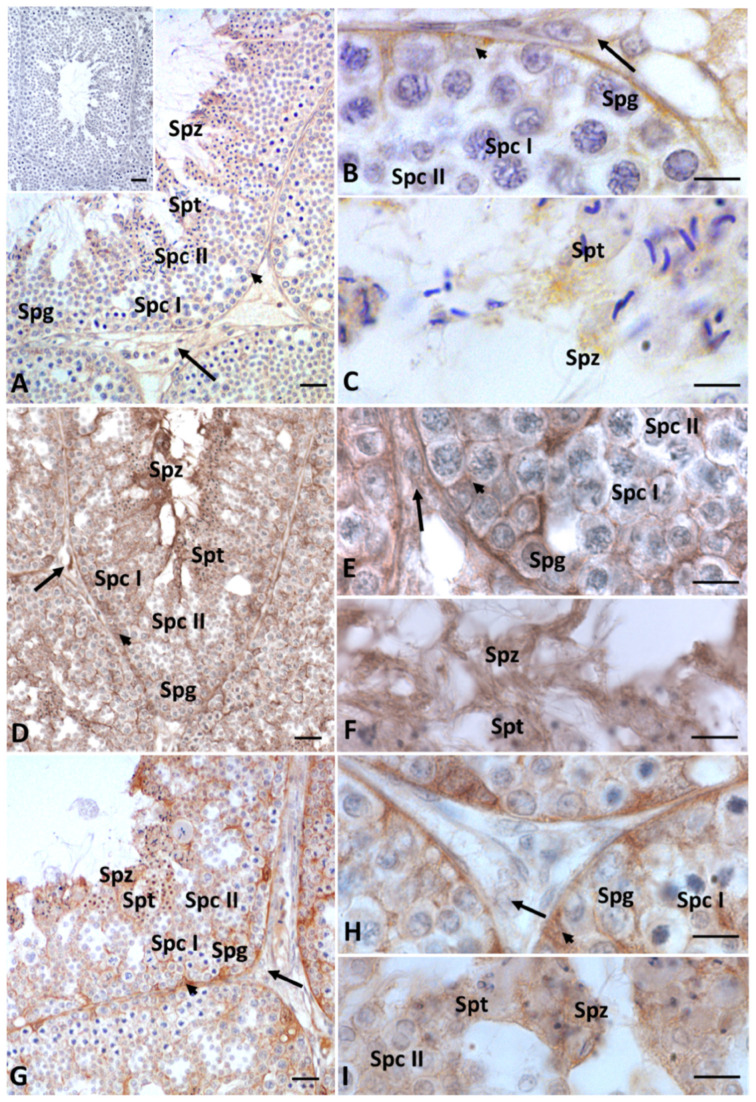
Immunohistochemical localization (brown areas) of ERβ in testis of *P. siculus* treated with different concentrations of glyphosate. (**A**–**C**) Control. The ERβ signal is evident in Sertoli cells (arrowhead), spermatids (Spt) and spermatozoa (Spz). (**D**–**F**) Low dose. Positivity is evident in Sertoli cells (arrowhead), spermatogonia (Spg), spermatocytes I (Spc I), spermatids (Spt) and spermatozoa (Spz). (**G**–**I**) High dose. A strong and wide positivity is evident in Sertoli cells (arrowhead), whereas a faint signal is present in spermatogonia (Spg), spermatocytes I (Spc I), spermatids (Spt) and spermatozoa (Spz). (**A**, insert): no signal is present in control sections. Scale bars correspond to 5 µm in figure (**B**,**C**,**E**,**F**,**H**,**I**); 20 µm in figures (**A**,**D**,**G**); and 50 µm in figure (**A** insert).

In the testes treated with HD Gly, the presence of Cx43 was significantly reduced, with the protein restricted to very few areas of the seminiferous epithelium (Figure 4C). In the testis of the control group animals, immunopositivity for 3-β-hydroxysteroid dehydrogenase (3β-HSD) and 17-β-hydroxysteroid dehydrogenase (17β-HSD) (data not shown), as well as for P450 aromatase, was recorded in both germline and somatic cells, as expected [33,34] (Figure 5A–C). Under normal conditions, estrogen receptors (ERs) showed a more restricted distribution in the testis. In particular, a faint signal for ERα was detected in spermatocytes I and II, spermatids and spermatozoa; a weak signal was recorded in Sertoli cells, whereas no Erα positivity was recorded in the other Leydig cells and spermatogonia (Figure 6A–C). As for ERβ, only spermatids, spermatozoa and Sertoli cells showed immunopositivity (Figure 7A–C).

After Gly treatments, no changes in the distribution of steroidogenic enzymes were recorded, regardless of dose (Figure 5D–I); on the contrary, the distribution of estrogen receptors showed some changes, especially ERβ. In particular, in the testis of both groups of lizards treated with Gly, we also detected the presence of ERα in spermatogonia and Leydig cells (Figure 6D–I) and of ERβ in spermatogonia and spermatocytes (Figure 7D–I); for the latter, a more diffuse signal was recorded within Sertoli cells and in spermatozoa in the LD-treated and especially in the HD-treated testis (Figure 7D–I) compared to those of the control animals (Figure 7A–C). For all the immunocytochemical analysis, no signal was detected by omitting the primary antibody.

### 2.3. Gene Expression of ERα and ERβ in Testis

**Figure 8:**
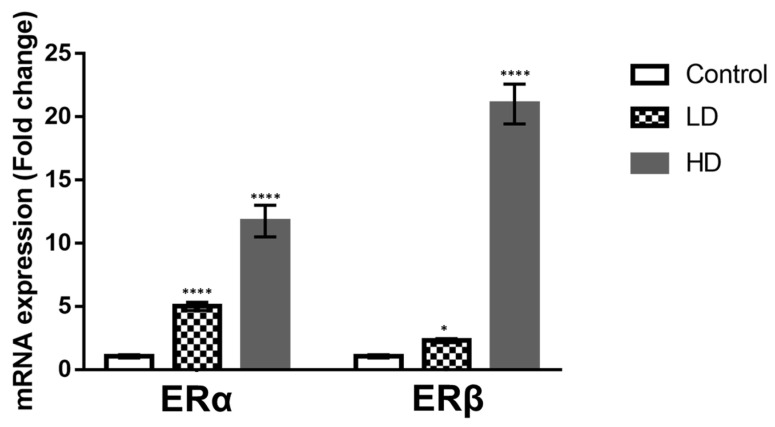
Real-time PCR for *P. siculus ERα* and *Erβ* mRNA. ERα/β actin and ERβ/β actin ratios are reported as fold change compared with the transcript level in control group. LD, low dose; HD, high dose. Real-time PCR analysis was performed in triplicate on five animals for each group. Bars represent the mean ± SD. Differences among the two treatments were statistically significant. * *p* < 0.05; **** *p* < 0.0001.

Changes in ERα and ERβ localization after Gly treatments prompted us to investigate the ER expression levels in the testes using real-time PCR analysis. The results show that Gly was able to elicit the expression of both estrogen receptors; *ERα* expression increased in a dose-dependent manner, *ERβ* significantly increased after HD Gly treatment (Figure 8). *ERα* transcripts increased by about 5-fold after LD treatment and by about 10-fold after HD treatment; *ERβ* expression was slightly upregulated in the animals treated with the lower Gly dose and (about 1-fold) and considerably increased following administration of the higher Gly dose (about 20-fold).

## 3. Discussion

In this study, we demonstrated that glyphosate treatment leads to alteration of spermatogenesis in a dose-dependent manner. In particular, morphological data showed a reduction in spermatozoa production, especially in the HD group, in which the epithelium of the seminiferous tubules showed many empty spaces and germ cells forming rosette-shaped aggregates. This result was supported by morphometric measurement, which showed an increase in the seminiferous tubules area in animals treated with a high dose of glyphosate compared to both the control and low dose groups, as well as more connective tissue in the intertubular areas. Picrosirius red staining highlighted the presence of fibrotic tissue in the interstitial areas due to excessive deposition of collagen, confirming the increase in connective tissue recorded during morphometric analysis. These results are in agreement with literature data, which report epithelial reduction and lumen enlargement in the seminiferous tubules of birds exposed to glyphosate [36]. Gly-induced tissue inflammation, abnormal collagen deposition and fibrosis formation have also been detected in mammalian tissues and in lizard liver [49,51,52,53]. The presence of aggregates of spermatocytes and empty spaces between germ cells prompted us to carry out an immunocytochemical investigations to evaluate the distribution of Connexin 43, a gap-junction protein responsible for Sertoli–Sertoli and Sertoli–germ cell communication [54]. Data described here demonstrate a dose-dependent reduction in the presence and distribution of Cx43 after treatment with Gly. In the control testes, Cx43 was present throughout the seminiferous tubule for the entire length of the Sertoli cells; in the samples treated with the low concentration of Gly, Cx43 was localized only in a few areas of the tubule, and a dramatic drop in Cx43 distribution was recorded with the high dose of glyphosate, with anti-Cx43 positivity reduced to a few spots. This result is in agreement with our morphological observations, as it is known in the literature that the interaction between Sertoli cells and germ cells up to the spermatids stage is fundamental to ensure the synchronous differentiation of germ cells and the regular distribution of these cells from the basal to the luminal part of the seminiferous tubules [55]. A recent in vitro study performed on cells isolated from prepubertal rat seminiferous tubules described a significant reduction in the number of germ cells, inhibition of clusterin expression, and no changes in Cx43 or occludin expression in the Sertoli cells after Gly treatments; this led the authors to hypothesize that the main target of Gly would be the protective role of Sertoli cell rather than cell junctions of the blood–testis barrier [56]. Our data, however, agree with Liu et al. who reported a loss of blood–testis barrier integrity in Gly-treated rats, which was associated with a significant downregulation of the expression of genes encoding both tight-junction (Occludin, Claudin 1) and gap-junction (Cx43) proteins.

Based on the observation that Connexin 43 expression in the testis, as well as in glioma cells, is under the control of estrogen/estrogen receptor signaling [57,58], one of the main pathways involved in the control of testicular activity [59,60], we decided to investigate any changes in the localization and expression of steroidogenic enzymes and estrogen receptors to clarify the possible endocrine-disrupting effect of glyphosate. Immunocytochemical investigations showed no change in the distribution of enzymes involved in the production of sex hormones, such as 3β-HSD, 17β-HSD and P450 aromatase, in Gly-treated samples, regardless of the dose used, compared to the control. A marked increase in *P450 aromatase* transcript and protein was reported in GBH-treated rat testis, but in this case, the treatment was carried out for a longer period (68–122 days) [31]. On the other hand, our data show a change in the distribution of estrogen receptors, in particular ERβ, in seminiferous tubules of Gly-treated lizards. We recorded a broader distribution of ERs in both low- and high-dose treatments; for ERβ, stronger labeling was observed in Sertoli cells and in late-stage germ cells. These data were supported by real-time PCR analysis, which demonstrated increased levels of both *ERα* and *ERβ* transcripts in treated samples compared to controls. Deregulation of the estrogen pathway by glyphosate has been extensively documented, albeit with contradictory results. In breast cancer cell lines, for example, Gly has been shown to activate ERs, likely through an indirect mechanism, being structurally incapable of binding ERα, as the molecular structures of Gly and estrogen are profoundly different [61]. Other authors instead suggested that Gly mimics estrogenic activity through interaction with its two receptors, ERα and ERβ, because ER antagonists block the action of glyphosate [62]. They found that Gly brought about up to 50% of the estrogenic response and induced breast cancer cell proliferation, as well as the expression of both ERs after 6 h and of only ERα after 24 h of exposure; however, in the presence of endogenous estrogen, this compound acted as an antagonist. In light of these results, it is conceivable that in lizards, Gly may act as an endocrine disruptor of the estrogen pathways, and this could explain the changes in ER expression recorded in our treatments. These changes could in turn lead to the reduced expression of Connexin 43, which is responsible of the structural alterations recorded in seminiferous tubules.

In this work, we described, for the first time, the toxic effects of glyphosate on the reproductive capacity of a lizard living in the ecosystem represented by fields and crops, on which it exerts a protective action against harmful insects. However, further investigations will be needed to verify the effect of glyphosate on testis functions at different concentrations and/or exposure times and to evaluate any synergistic effects on these functions of various contaminants to which these animals may be exposed. In this context, it is important to underline that various pollutants can alter the functionality of the male gonad; recently, it was shown that mercury, a toxic metal, in addition to modifying steroidogenesis and gene expression in the male gonad of *Mytilus galloprovincialis*, makes spermatozoa more susceptible to the action of the micrococcal nuclease, and this is indicative of an improper sperm chromatin structure [63].

## 4. Conclusions

Although many attempts have been made to answer the question of whether exposure to Gly endangers animals, the issue remains unsolved due contradictory results reported in the literature. It has been suggested that Gly has endocrine-disrupting properties, resulting in a negative impact on male reproduction. Even less well known are the effects of Gly on wildlife. In this context, we demonstrated that exposure of lizards to Gly resulted in an alteration of the morphology of the seminiferous tubules, mainly due to disruption of the gap junction between cells, as well as in changes in the testicular expression of estrogen receptors. These effects are mostly dose-dependent, indicating that the herbicide can cause disturbance in the morphophysiology of the male lizard’s reproductive system, with obviously detrimental effects on their reproductive fitness. Putting together the Gly-induced liver injuries previously observed in this lizard [49] and the testicular damage described in this work, we can state that accidental exposure to glyphosate, which may be quite common in animals inhabiting crop fields, gardens and parks, can endanger the survival of this species. Furthermore, by highlighting the cytotoxic effects of oral exposure to Gly on male gametogenesis in a terrestrial vertebrate, the data also suggest the importance of promoting agricultural practices that discourage its use or otherwise limit its concentration.

## 5. Materials and Methods

### 5.1. Animals and Experimental Design

Sexually mature male specimens of *Podarcis siculus* (about 7 cm snout-vent, 10 g body weight) were captured near Naples (Campania, Italy, Latitude: 41°19′54″ N; Longitude: 13°59′29″ E) in late October. Lizards were maintained in soil-filled terrariums at natural temperature (about 20 °C) and photoperiod and fed ad libitum with *Tenebrio molitor* larvae. After 7 days of acclimatization, they were randomly divided into three groups (*n* = 10); lizards of group 1 and 2 were exposed to pure Gly at doses of 0.05 (low dose, LD) and 0.5 (high dose, HD) μg/kg body weight, respectively, in 50 µL tap water via oral gavage every other day for 3 weeks; animals of group 3 (control) received, by gavage, the same doses of tap water. Animals were exposed to pure Gly to exclude a possible interference of the adjuvants. These experimental doses correspond to 104× and 103× acceptable daily intake (ADI) relative to the European ADI in mammals [64]. At the end of treatments, all the animals were killed by decapitation after deep anesthesia with ketamine hydrochloride (Parke-Davis, Berlin, Germany), 325 μg/g body weight. Testes were immediately excised; for each animal, one testis was fixed in Bouin for immunohistochemical investigation, histological and morphometric analysis; the other was processed for biomolecular analysis.

The experiments were organized to minimize stress and the number of animals used. According to the 3 Rs (replacement, reduction and refinement) principle, to maximize the information obtained per animal and thus limit the subsequent use of additional animals, the lizards employed in this study were the same as those used in our previous study on glyphosate.

### 5.2. Histological and Morphometric Analysis

Testes fixed in Bouin’s fluid were dehydrated in a graded series of alcohols, clarified in xylene and embedded in paraffin. Testis sections (7 μm thick) were dewaxed, rehydrated and stained with Mallory’s trichrome and Picrosirius red mixture. Randomly chosen sections (*n* = 3) from each animal (*n* = 5) were digitized using LAS EZ software. Thirty tubules for randomly selected areas of different histological sections were analyzed. Histological structures (both germinal epithelium and connective tissue) were measured with ImageJ software.

### 5.3. Immunoistochemistry

Immunohistochemistry investigations were performed as previously reported [65]. For antigenic unmasking, 5 µm thick sections were treated with 10 mM citrate buffer with a pH of 6.0 and then incubated in 2.5% H_2_O_2_ in methanol for endogenous peroxidase blocking. The non-specific background was reduced with incubation in normal goat serum (Pierce, Rockford, IL, USA) for 1 h at room temperature. Slides were then incubated overnight at 4 °C with the following primary antibodies diluted in normal goat serum: rabbit anti-mouse Cx43 (1:300), rabbit anti-human 3β-HSD (1:100), rabbit anti-mouse 17β-HSD (1:100), rabbit anti-human P450 aromatase (1:200), rabbit anti-human ERα (1:200) and rabbit anti-ERβ (1:200). All the antibodies were from Santa Cruz Biotechnology (Santa Cruz, CA, USA), except anti-mouse Cx43, which was from Elabscience (Houston, TX, USA).

The next day, sections were incubated with HRP-conjugated goat anti-rabbit or mouse secondary antibody diluted to 1:200 in normal goat serum for 1 h at room temperature and then incubated with avidin–biotin–peroxidase complex (ABC immune peroxidase kit, Pierce) for 1 h at room temperature. Finally, sections were stained using diaminobenzidine (DAB) as chromogen and counterstained with Meyer’s hematoxylin. Negative controls were processed by omitting incubation with primary antibody.

The immunohistochemical signal was observed using a Zeiss Axioskop microscope, and the images were acquired using Axiovision 4.7 software (Zeiss).

### 5.4. RNA Isolation and Real-Time Polymerase Chain Reactions

Total RNA of good quality (260/280 nm ratio = 1.9) was extracted individually from each testis using TRIzol reagent (ThermoFisher Scientific, Waltham, MA, USA) according to the manufacturer’s instructions. Removal of genomic DNA and single-strand cDNA synthesis were performed using a QuantiTect reverse transcription kit (Qiagen, Hilden, Germany) according to the manufacturer’s protocol. Real-time PCR reactions were carried out in triplicate for each sample with an Applied Biosystems 7500 real-time system by and Power SYBR Green master mix PCR (Applied Biosystems, Waltham, MA, USA), following the procedures recommended by the manufacturer. β-actin gene expression was used as an internal standard control. Specific primers for *P. siculus* ERα, ERβ and β-actin genes were designed as previously reported [45,66]. Changes in gene expression relative to the different samples were calculated according to the standard 2^−ΔΔ^Ct method described by Livak and Schmittgen [67]. Data are presented as mean ± standard error of the mean (SEM) from three separate experiments in each sample (*n* = 6).

### 5.5. Statistical Analysis

Statistical analysis was performed using GraphPad Prism 8 software. The statistical significance was calculated using one-way ANOVA followed by Bonferroni’s multiple comparison test, and differences were considered statistically significant when *p* < 0.05.

## Data Availability

The authors confirm that the data supporting the findings of this study are available within the article.
